# Role of Cell Death in Cellular Processes During Odontogenesis

**DOI:** 10.3389/fcell.2021.671475

**Published:** 2021-06-18

**Authors:** John Abramyan, Poongodi Geetha-Loganathan, Marie Šulcová, Marcela Buchtová

**Affiliations:** ^1^Department of Natural Sciences, University of Michigan–Dearborn, Dearborn, MI, United States; ^2^Department of Biological Sciences, SUNY Oswego, Oswego, NY, United States; ^3^Department of Experimental Biology, Faculty of Science, Masaryk University, Brno, Czechia; ^4^Laboratory of Molecular Morphogenesis, Institute of Animal Physiology and Genetics, Czech Academy of Sciences, Brno, Czechia

**Keywords:** teeth, dental lamina, apoptosis, odontogenesis, morphogenesis

## Abstract

The development of a tooth germ in a precise size, shape, and position in the jaw, involves meticulous regulation of cell proliferation and cell death. Apoptosis, as the most common type of programmed cell death during embryonic development, plays a number of key roles during odontogenesis, ranging from the budding of the oral epithelium during tooth initiation, to later tooth germ morphogenesis and removal of enamel knot signaling center. Here, we summarize recent knowledge about the distribution and function of apoptotic cells during odontogenesis in several vertebrate lineages, with a special focus on amniotes (mammals and reptiles). We discuss the regulatory roles that apoptosis plays on various cellular processes during odontogenesis. We also review apoptosis-associated molecular signaling during tooth development, including its relationship with the autophagic pathway. Lastly, we cover apoptotic pathway disruption, and alterations in apoptotic cell distribution in transgenic mouse models. These studies foster a deeper understanding how apoptotic cells affect cellular processes during normal odontogenesis, and how they contribute to dental disorders, which could lead to new avenues of treatment in the future.

## Introduction

Over the past several decades, the contribution of apoptosis to vertebrate odontogenesis has received considerable attention, promoted by technical advancement and increased availability of diverse laboratory models (Nozue, 1971; Moe and Jessen, 1972; Kindaichi, 1980; Nishikawa and Sasaki, 1995; Shibata et al., 1995; Lesot et al., 1996; Vaahtokari et al., 1996). More recent studies have revealed that apoptosis is not just a silent mechanism of cell removal during embryonic development. Rather, apoptotic cells produce numerous signaling molecules that affect the behavior of surrounding cells, inducing morphogenesis, cell migration, and alteration of cell fate. Here, we review these relationships and propose a broader contribution of apoptosis to the cellular processes of odontogenesis than was previously thought. First, we briefly summarize the distribution of apoptotic cells during mammalian odontogenesis since mammals represent the most common models for the study of their localization, distribution, and function during odontogenesis. Next, we review the available literature on non-mammalian groups such as reptiles and fishes, which are becoming increasingly common models for the study of odontogenesis due to their unique dental characteristics. Subsequently, we discuss cellular processes directed by the effects of apoptosis on surrounding non-apoptotic cells, as well as the indirect roles of apoptosis in individual steps of tooth development and morphogenesis.

## Distribution of Apoptotic Cells During Odontogenesis in Vertebrates

Tooth development is characterized by complex, reciprocal interactions between the stomodeal epithelium and the underlying cranial neural crest-derived mesenchyme (Thesleff, 2003; Balic, 2019). This interaction drives tooth morphogenesis, including differentiation of individual components at the molecular level (Thesleff, 2003; Balic and Thesleff, 2015). In spite of differences in final size and shape, teeth undergo consecutive developmental stages common to all vertebrates, including epithelial thickening, bud, cap, and bell stages (Thesleff, 2003). The distribution of apoptotic cells during odontogenesis strongly correlates with specific morphogenetic events and associated tissues. For example, apoptosis is usually confined to epithelial cells undergoing folding, while very few dying cells are found in the surrounding mesenchyme. We first review the localization of apoptotic cells in mammalian dentition, focusing on the mouse as the most common model organism, and then compare their distribution to non-mammalian groups.

### Mammalia

**FIGURE 1:**
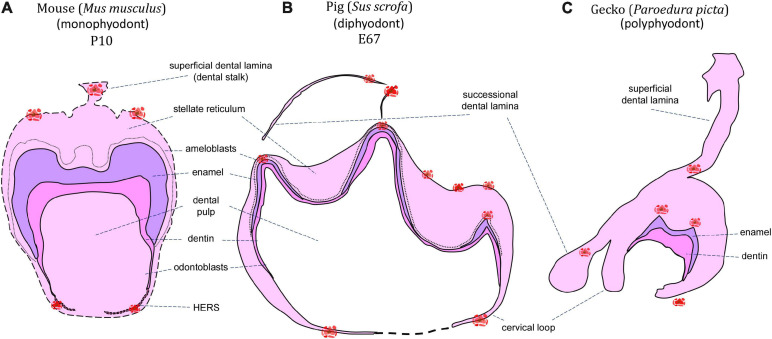
Schematic of apoptotic cells distribution through mineralization stage of the tooth germ. Clusters of apoptotic bodies are labeled in red and label areas of possible future interest in monophyodont mouse **(A)**, diphyodont pig **(B)**, and polyphyodont gecko **(C)**.

At early developmental stages, apoptotic cells are located in the budding epithelium of the molars, specifically in the cells facing the oral cavity (Peterková et al., 1998). After epithelial invagination, a streak of apoptotic cells extends to the tip of the developing molar bud (Nair, 2010). At the cap stage, clusters of apoptotic cells in the inner enamel epithelium localize to the primary enamel knots (PEKs), as was shown by studies in the murine molar (Lesot et al., 1996; Jernvall et al., 1998). Later, at the bell stage of molar development, apoptosis can be detected in the secondary enamel knots (SEKs) and surrounding cells, including the stratum intermedium and adjacent mesenchyme (Vaahtokari et al., 1996). After the disappearance of the enamel knots, apoptotic cells appear in the superficial part of the dental lamina (Lungová et al., 2011), which develops in mouse as just a short epithelial connection between the tooth germ and the oral epithelium, sometimes called the dental stalk or the gubernaculum (Dosedelova et al., 2015; Chaudhry and Sobti, 2020). After the molar is fully formed, apoptotic cells are also involved in the tooth eruption stage, exhibiting concentrations in the oral epithelium above the erupting teeth as well as the superficial part of the dental lamina (Moriguchi et al., 2010; Dosedelova et al., 2015; Figure 1). Apoptotic cells are also situated in the anterior-most portion of the first molar epithelium (Lesot et al., 1996), which in rodents abuts an edentulous diastema. Interestingly, the mandibular diastema is devoid of apoptosis, whereas the maxilla displays apoptotic signal in the diastema region, attributed to the presence of transitory tooth buds in the maxillary diastema (Vaahtokari et al., 1996). At the bud stage, there are no apoptotic cells seen in the mesenchyme (Nair, 2010). In later developmental stages during root formation, mesenchyme exhibits apoptosis linked with a proportion of the Hertwig’s epithelial root sheath (HERS) cells, as was shown in the rat upper molar (Kaneko et al., 1999). Remaining sheath cells aggregated in the periodontal area and form the epithelial rests of Malassez (ERM) (Hamamoto et al., 1989), which also undergo apoptosis later in development as part of a normal mechanism of turnover or remodeling (Cerri and Katchburian, 2005; Lee et al., 2012).

Apoptotic cells, identified as osteoclasts, are also located on the surfaces of the developing alveolar bone around developing molars (Vaahtokari et al., 1996). Growing teeth have previously been shown to be associated with osteoclast activity and resorption of the surrounding alveolar bone in embryonic mice (Reponen et al., 1994), or the remodeling of bone due to compressive forces produced by the occlusion and eruption of ever-growing rodent incisor (Irie and Ozawa, 1990). Apoptosis may serve to remove these osteoclasts after they have completed their function of creating space/facilitating interaction between the tooth and surrounding bone. In support of this theory, the elimination of compressive forces by the incisors has been found to lead to the inactivation of osteoclasts (Irie and Ozawa, 1990). Otherwise, there are surprisingly few apoptotic cells located in the odontogenic mesenchyme, and those present are without any discernable pattern (Stock et al., 1997). Why are there so few apoptotic cells in the mesenchyme? One possible explanation is the considerable plasticity of the mesenchyme during odontogenesis, where cells can be easily relocated in the loose tissue architecture without the necessity of eliminating cells through death. Moreover, cells spread throughout the dental papilla and dental follicle, with cell signaling forming gradients without any local concentration or the presence of distinct signaling centers.

It should be noted that in rodents, the above-described patterns only apply to molar development, with the incisors exhibiting a different pattern of apoptosis. In mice, the epithelial thickenings that initiate incisor development demonstrate low numbers of apoptotic cells (Kieffer et al., 1999). Later, apoptotic cells can be found in the superficial part of the dental lamina connecting the enamel organ with the oral epithelium (Lesot et al., 1996; Kieffer et al., 1999), as well as in the inner dental epithelium close to the epithelio-mesenchymal junction at the future incisor ridge (Kieffer et al., 1999). Interestingly, an accumulation of apoptotic cells is also found in the mesenchyme surrounding the labial cervical loop, in the area where development of the cervical loop was more pronounced compared to the lingual side (Kieffer et al., 1999).

### Reptilia

**FIGURE 2:**
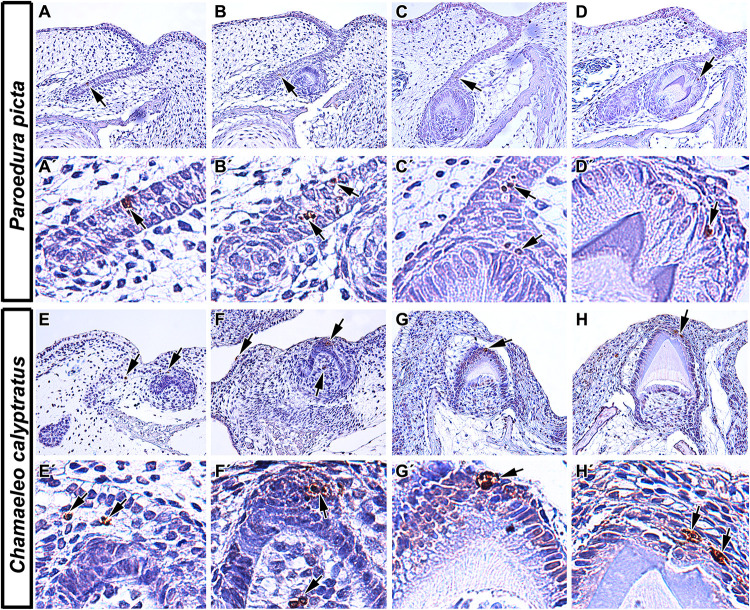
Localization of apoptotic cells in squamate teeth. **(A–D)** Detection of apoptotic cells in developing tooth germs of Ocelot gecko (*Paroedura picta*). **(A,A′,B,B′)** TUNEL-positive cells were found both in the epithelium of interdental dental lamina and in successional dental lamina, indicating their role not only in the growth of tooth germs but also in continuous tooth replacement (black arrows). **(C,C′)** Apoptotic cells were also situated in the stellate reticulum of the developing tooth and in the EK-like cluster of cells (black arrows). **(D,D′)** Later in development, TUNEL-positive cells were mostly situated above the enamel ridge (black arrow). **(E–H)** Presence of TUNEL positive cells during different stages of odontogenesis in Veiled chameleon (*Chamaeleo calyptratus*). **(E)** Apoptotic cells appear in the presumptive stellate reticulum of early cap stage tooth germ **(E′)** and in the labially situated, developing salivary glands. **(F,F′)** Later, clustering of TUNEL positive cells takes place in the EK-like area of the cap stage tooth germ, with few apoptotic cells found in the adjacent mesenchyme **(F)**. **(G,G′,H,H′)** Once odontogenesis proceeds and hard tissue production has begun, there are a few TUNEL-positive cells located at the top of the forming ridge, delimiting the margins of developing enamel grooves. TUNEL-positive cells (brown, DAB), TUNEL-negative cells (blue, Hematoxylin).

The presence of apoptotic cells in the context of tooth development has been previously described in a several species of crocodilians and squamates (snakes and lizards). A key reason for the use of reptiles as models for odontogenesis is the fact that most species are polyphyodont and exhibit lifelong tooth replacement, with a smaller subset being monophyodont and developing a single generation of teeth that fuse to the jaws and are never replaced (Edmund, 1960; DeMar, 1972). During reptile odontogenesis, apoptosis is associated with the enamel knot region (homologous to the mammalian enamel knots) at early developmental stages, and the formation of complex tooth morphology at later mineralization stages. Additionally, apoptosis may be found during successional dental lamina development/disruption, egg tooth formation or venom canal formation in viperid snakes (Figures 1, 2).

At early – cap and bell – stages, apoptotic bodies were detected in the enamel organ of several reptile species including veiled chameleon (*Chamaeleo calyptratus*), ocelot gecko (*Paroedura picta*), bearded dragon (*Pogona vitticeps*), African rock python (*Python sebae*), and Siamese crocodile (*Crocodylus siamensis*) (Buchtová et al., 2007; Handrigan and Richman, 2010; Landova Sulcova et al., 2020). The veiled chameleon possesses heterodont teeth, with the rostral-most teeth being nearly conical with one rounded tip, whereas more caudal teeth are multiple-cusped, with a dominant central cusp flanked by accessory cusps (Landova Sulcova et al., 2020). Additionally, the central cusp is divided into labial and lingual crests, separated by a shallow groove. In the chameleon (Figure 2), the pattern of apoptotic cell distribution is similar to what was described in the mammalian tooth, with apoptotic cells located in an enamel knot-like cluster at the cap stage (Landova Sulcova et al., 2020), revealing the existence of a similar signaling center as was described in mammals. The ocelot gecko is a homodont species that has small, peg-shaped teeth with labial and lingual enamel crests at the tips, similar to the central cusp of the chameleon tooth (Landova Sulcova et al., 2020). Developing gecko teeth also exhibit recognizable enamel knot-like structures with few apoptotic cells located in the inner enamel epithelium or in the stellate reticulum just above it (Landova Sulcova et al., 2020). Later, at the bell stage, apoptotic cells are situated at the tip of the inner enamel epithelium where morphogenesis occurs, and where the future cusp will form.

In bearded dragon, which has broad, triangular and single-cusped teeth (Handrigan and Richman, 2011), apoptosis was found in the dental papilla, odontoblasts, and pre-ameloblasts of superficial (vestigial) teeth (Handrigan and Richman, 2010). When the developing tooth germ was exposed to cyclopamine, a proven SHH inhibitor, an increased number of apoptotic cells appeared in the stellate reticulum (Handrigan and Richman, 2010) using a TUNEL (Terminal deoxynucleotidyl transferase dUTP nick end labeling) assay, confirming the role of SHH in cell maintenance. TUNEL assays are one of the most common methods for the detection of apoptotic cells by targeting DNA fragmentation during programmed cell death (Gavrieli et al., 1992). Nevertheless, the possibility of toxicity from cyclopamine should be considered in this case. In the African rock python, apoptosis was also detected during the early stages of tooth development, localized in the stellate reticulum of the enamel organ. However, an obvious morphological appearance of enamel knot-like structure was not observed in this species (Buchtová et al., 2007). The absence of a morphologically distinct enamel knot is usually associated with the development of a very small enamel organ and reduced stellate reticulum. However, the existence of an apoptotic cell cluster in the same area as observed in other reptile species may indicate an identical function for apoptosis during odontogenesis in python.

It is important to note that while some reptile species exhibit characteristics of mammalian enamel knots such as reduced proliferation, specific expression of SHH, FGF, and BMP signaling molecules, and apoptosis (Jernvall et al., 1998), many do not. For example, thickened dental epithelium has previously been described for the American alligator (*Alligator mississippiensis*) (Westergaard and Ferguson, 1987), veiled chameleon (Buchtová et al., 2013) as well as the leopard gecko (*Eublepharis macularius*) (Handrigan and Richman, 2011). Meanwhile histological studies of ball python (*Python regius*) and the bearded dragon revealed a distinct lack of any such tissue swelling (Handrigan and Richman, 2011). Furthermore, within those with an “enamel knot” homolog such as the chameleon and ocelot gecko, some of the classic enamel knot characteristics such as reduced proliferation, SHH expression, and even apoptosis were observed (Landova Sulcova et al., 2020), while reptiles with simple, unicuspid teeth such as snakes, exhibit no apoptosis in the inner enamel epithelium but a cluster of TUNEL-positive cells were located in the stellate reticulum just above (Buchtová et al., 2008). Therefore, there does appear to be a signaling center similar to the mammalian enamel knot in some reptile lineages, but its development seems to be associated with the general complexity of tooth crown morphology.

An additional key difference of reptilian odontogenesis is their enhanced tooth replacement. The fact that polyphyodont and monophyodont species have been studied, and that the retention of a successional lamina is the key difference between the two groups, we can now obtain a better appreciation for how apoptosis separates the two categories of reptiles. Apoptotic cells have been identified in association with the dental lamina in reptiles regardless of tooth generation number. However, there are some differences in their distribution and amount of apoptotic cells depending on the length of time for dental lamina persistence. In polyphyodont species, where an extensive dental lamina connects several tooth generations, a successional lamina is retained as an extension of the dental lamina off the newest forming tooth and facilitates continuous replacement of teeth (Whitlock and Richman, 2013). In diphyodont species, the dental lamina degenerates during the initiation of the second tooth generation (Whitlock and Richman, 2013). In the veiled chameleon, a monophyodont species, a successional lamina is initiated during embryonic development but later regresses. Surprisingly, even the degenerating dental lamina exhibited very few apoptotic cells, with removal attributed to different cellular mechanisms (Buchtová et al., 2013). In the bearded dragon, another monophyodont species, apoptosis was located in the degrading dental lamina in association with decreased WNT pathway activity (Richman and Handrigan, 2011). However, a subsequent study found just a few TUNEL-positive cells under normal physiological conditions, and those were restricted to the mesenchyme surrounding the successional dental lamina (Salomies et al., 2019). Since lamina morphology differs along the jaw in this species, this discrepancy in apoptotic cell numbers could be explained by local differences between morphology and role of the dental lamina along the rostral-caudal axis of the jaw. In the polyphyodont ocelot gecko (Figure 2), a few TUNEL-positive cells were located in the dental lamina but not in its tip, but rather in a more superficial area at the edge of the enamel organ of the associated tooth (Landova Sulcova et al., 2020). In the African rock python, another polyphyodont species, numerous TUNEL-positive cells were found in the dental lamina connecting the tooth to the oral epithelium, but once again not in the tip of the successional lamina (Buchtová et al., 2007).

In reptiles, later mineralization stages of odontogenesis reveal apoptosis associated with the formation of an enamel groove. In veiled chameleon and ocelot gecko (Figure 2), both of which exhibit enamel ridges at the tooth tip with two crests and a central groove, identical distribution of TUNEL-positive cells was described. Once cell differentiation advanced and mineralization of enamel progressed, two distinct clusters of apoptotic bodies were detected at the margins of the developing enamel grooves. In contrast, the relatively simple, conical teeth of Siamese crocodiles possessed only one distinct apoptotic area at the very tip of the single cusp (Landova Sulcova et al., 2020).

Apoptosis is also crucial for the formation of the venom canal in the fangs of viperid snakes. In the white-lipped pit viper (*Trimeresurus albolabris*), apoptotic cells are located in the central area of the first developing fang, where they contribute to cell removal in the formation of an empty venom transport canal (Zahradnicek et al., 2008). Interestingly, apoptotic cells were not found during the early stages of canal invagination and do not seem to be responsible for the loss of inner enamel epithelium in the shaft area prior to the cell removal process. Besides concentration in the central canal, apoptotic cells are also situated in the tip of the fangs in two symmetrical lateral zones. It was proposed that these cells contribute to the clearance of space for the later emergence of the tooth from the oral mucosa, but the possible role in morphogenesis and cell arrangement in the inner enamel epithelium may also be a valid prediction here (Zahradnicek et al., 2008).

Apoptosis was also identified in the egg tooth of the brown anole (*Anolis sagrei*) (Hermyt et al., 2020). Since most reptiles are oviparous, they have evolved “egg teeth” that assist in their hatching from the amniotic egg. In some groups, a caruncle develops in the form of a modified scale (e.g., birds and turtles) (Clark, 1961), while squamates develop a structure that is for all intents and purposes, a tooth (Fons et al., 2020). In the brown anole, degeneration of the enamel organ was observed in the egg tooth shortly before its eruption and after subsequent hatching, and this process was attributed to apoptosis based on morphological characteristics (Hermyt et al., 2020). Authors theorized that loosening of the intercellular junctions in that epithelium, as opposed to simply shedding it, would allow for penetration of inflammatory cells and tissue exudate, making it a first-line of defense against pathogenic infections, similar to the junction epithelium in mammals (Hermyt et al., 2020). An earlier study from the same group identified degeneration of the egg tooth enamel organ shortly before hatching also in the grass snake (*Natrix natrix*) (Hermyt et al., 2017).

Several groups of reptiles develop embryonic (i.e., vestigial or superficial) teeth (Westergaard and Ferguson, 1986; Handrigan and Richman, 2010; Zahradnicek et al., 2012), the function of which remains a mystery. Superficial teeth of the American alligator (*Alligator mississippiensis*) develop from elevations along the oral epithelium, almost perpendicular to the jaw surface, before the formation of the dental lamina, and they are described as being poorly differentiated and lacking enamel (Westergaard and Ferguson, 1986). As embryonic development progresses and functional teeth start to form, the dental cells of the embryonic teeth begin to degenerate; leaving a dentine matrix that is either resorbed or shed. Westergaard and Ferguson hypothesized that there may be “death factors” at play here, however, they do not specifically show any evidence of apoptotic cells in their studies (Westergaard and Ferguson, 1986). In the bearded dragon, on the other hand, Handrigan and Richman performed TUNEL analysis and detected apoptosis in the dental papilla and pre-ameloblast cells as the vestigial tooth generation reached the cap stage of development (Handrigan and Richman, 2010).

### Amphibia

Living amphibians are classified into three Orders: frogs and toads (Order: Anura), salamanders and newts (Order: Caudata), and caecilians (Order: Gymnophiona). Despite being one of the most charismatic groups of vertebrates, there is little in the way of recent scientific literature focusing on the cellular processes during their odontogenesis. Early studies indicate that some form of cell death does take place during amphibian odontogenesis, with reference to “autophagy,” “necrosis,” and “degeneration” of cells, some of which are likely to be apoptotic events that have not been recognized as such.

Anurans are polyphyodont and develop simple, conical, bicuspid teeth during metamorphosis (Gillette, 1955; Shaw, 1979). Prior to odontogenesis, anuran larvae (tadpoles) possess keratinized mouthparts that function in the place of true teeth and that are broken down through autolysis at metamorphosis (Kaung, 1975; Davit-Beal et al., 2007). While there is little in the way of evidence for apoptosis during odontogenesis in anurans, several authors mention “degeneration” of cells at the tip of the enamel organ immediately prior to eruption (Shaw, 1979; Huysseune and Sire, 1998). In *Xenopus laevis*, an aquatic species from the family Pipidae, odontoblasts at the tips of the developing teeth were shown to flatten, change orientation, and nuclei become pyknotic just when metamorphosis is complete (Shaw, 1979); where pyknosis is now recognized as a telltale sign of apoptosis or necrosis in a cell (Burgoyne, 1999). The degenerative process was described as continuing into the basal part of the teeth until no active odontoblasts were visible (Shaw, 1979). In the leopard frog (*Rana pipiens*), a terrestrial species from the family Ranidae, Zaki, and MacRae identified autophagic vacuoles in ameloblasts, concomitant with the loss of some cells at the transitional stage of amelogenesis (Zaki and MacRae, 1977). These vacuoles were observed to contain debris of membranous organelles and were associated with lysosome-like structures, and therefore, were assumed to be autophagic. In a later study comparing secretory and non-secretory ameloblasts, they specify that the autophagic vacuoles were only found in secretory ameloblasts (Zaki and MacRae, 1978). However, the authors ultimately concluded that these structures are involved in reorganization rather than replacement of ameloblasts (Zaki and MacRae, 1977).

**FIGURE 3:**
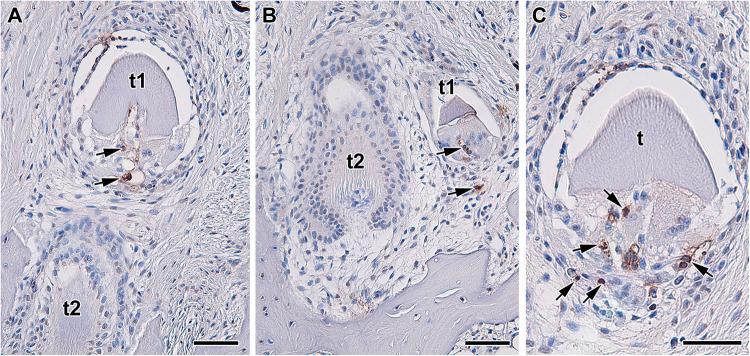
Localization of apoptotic cells during tooth replacement. **(A–C)** Apoptotic cells (arrows) are located in the dental papilla and underlaying area of dental follicle in teeth in regression. Apoptosis was labeled by TUNEL assay in transversal section through upper jaw of *Xenopus tropicalis* t1, t2 - teeth. TUNEL-positive cells (brown, DAB), TUNEL-negative cells (blue, Hematoxylin). Scale bar = 50 μm.

Salamanders and newts also undergo metamorphosis, albeit involving a less drastic morphological change than anurans. As such, odontogenesis of relatively simple, bicuspid teeth begins at larval stages, with a general transition from monocuspid to bicuspid teeth at metamorphosis (Davit-Beal et al., 2007). Since all amphibians are polyphyodont (Tucker and Fraser, 2014), much of the literature on cellular breakdown is focused on tooth replacement. In the Iberian ribbed newt (*Pleurodeles waltl*), during resorption of the first-generation teeth, necrosis was reported in several cell populations in the pulp cavity and HERS, in conjunction with osteoclasts (or odontoclasts) and macrophages (Davit-Beal et al., 2007). In the axolotl (*Ambystoma mexicanum*), the degeneration of odontoblasts and ameloblasts was described after ankylosis of the tooth to the jaw (Wistuba et al., 2002), although once again there is no specification of this being an apoptotic event.

### Actinopterygii

The zebrafish is the preeminent fish model in the field of odontogenesis, despite the lack of oral dentition and formation of only relatively simple, conical pharyngeal teeth associated with their rear branchial arches (pharyngeal jaws) (Wautier et al., 2001). Regardless of their simplicity and small size, pharyngeal teeth exhibit continuous replacement, as well as position-dependent differences in tooth length, height, neck–crown angle, cusp depth, and crown curvature, making them a useful model for odontogenesis in vertebrates (Wautier et al., 2001). However in zebrafish, apoptosis is not involved in odontogenesis to the extent as was observed in tetrapods. At early stages, no apoptotic cells were found in the dental epithelium and only a few Caspase3-positive cells were detected in the mesenchyme adjacent to the tooth germ, indicative of apoptosis (Yu et al., 2015). At later developmental stages, apoptotic cells were located in the distal part of the dental epithelium at 72 hours post-fertilization (hpf) in the area where the tip of the tooth is formed. This distribution of apoptotic cells is similar to reptile species with very small and simple-shaped tooth crowns. The question of whether there is a larger involvement of apoptotic cells in the dental epithelium of more complex teeth in different fish species will be interesting to pursue.

Even when the odontogenic pathway is disrupted, little apoptosis is induced in zebrafish. For example, when the SHH signaling pathway was perturbed by CyA treatment, morphological changes in the tooth germ were observed, specifically associated with the reduction of the dental papilla (Yu et al., 2015). Numerous Caspase3-positive cells were identified in the dental epithelium and mesenchyme surrounding tooth germ at early and later stages, which is indicative of apoptosis. Surprisingly, there were no apoptotic cells found inside the dental papilla, which was strongly affected. Therefore, apoptosis does not seem to contribute to dental papilla reduction and other cellular mechanisms that cooperate to augment shape changes after SHH inhibition.

In non-model fish, there is also little evidence for apoptosis during odontogenesis. In gar embryos, which develop single-cusped, conical oral dentition, the expression of protein p63 was detected in almost the entire dental epithelium (Rostampour et al., 2019). Activation of p63 protein in so-called TA form (bearing a transcription activation domain) usually leads to transcription of genes resulting in cell cycle arrest or apoptosis (Little and Jochemsen, 2002). However, the association of p63-positive cells to apoptosis remains to be investigated.

### Chondrichthyes

In the catshark (*Scyliorhinus canicula*), which possesses teeth with a long central cusp and various numbers of lateral smaller cups, TUNEL analysis failed to uncover apoptotic cells during early odontogenesis, or later during tooth shaping, leading to the conclusion that there is no true enamel knot in Chondrichthyes (Debiais-Thibaud et al., 2015). The murine enamel knot is generally defined as a non-proliferative, tightly packed group of cells that express SHH, FGF, and BMP signaling molecules, that finally meet an apoptotic fate (Jernvall et al., 1998). However, despite some differences in histological appearance, studies have identified homologous regulatory pathways (SHH, BMP and FGF) (Debiais-Thibaud et al., 2015; Rasch et al., 2016), as well as a distinct lack of proliferation signaling in dental epithelium (Rasch et al., 2016), suggestive of a homologous signaling center if not a true enamel knot (Rasch et al., 2016). The lack of an enamel knot may also be associated with the fact that sharks do not form true enamel, instead producing an enamel-like, mineralized tissue called enameloid (Gillis and Donoghue, 2007; Manzanares et al., 2016). Such findings in non-mammalian research models will likely redefine exactly what the field refers to as an “enamel knot” and whether the apoptotic end of the mammalian enamel knot should be considered a defining characteristic.

## Roles of Apoptosis in Morphogenetic Processes During Odontogenesis

Previous studies have proposed a number of roles for apoptosis during odontogenesis, including the shaping of embryonic structures through selective deletion of specific cells or cell populations. During odontogenesis, clusters of apoptotic cells may be found in key positions that contribute to the morphology of the tissue. Here, we review different morphogenetic processes where apoptosis is involved.

### Apoptosis in Epithelial Invagination During Early Odontogenesis

While there are no apoptotic cells in the epithelial thickening of the oral epithelium, later in development (E12–E13 mouse embryo), during epithelial budding, apoptotic cells are localized to the oral surface in the central area of the tooth bud, as well as in the budding epithelium just beneath the oral ectoderm (Peterková et al., 1996; Vaahtokari et al., 1996). This localization of apoptotic cells suggests possible involvement of cell death in budding morphogenesis in the epithelium. One possible mechanism for this process may be the induction of epithelial bending through cytoskeletal rearrangement and generation of an apicobasal pulling force, which induces deformation of the surrounding cells and the epithelial surface (Monier et al., 2015). In mouse, phalloidin staining has confirmed uneven distribution of actin filaments in epithelial thickenings during early molar development (Li et al., 2016). Cytoskeleton rearrangement associated with apoptotic cell appearance has also been previously observed at later stages of odontogenesis, during inner enamel epithelium folding and enamel ridge formation in chameleon (Landova Sulcova et al., 2020). Therefore, it is probable that clusters of apoptotic cells observed during epithelial budding produce a pulling force generated by their accumulation in a small, localized area and thereby, contribute to the folding of surface epithelium during invagination.

### Inhibition of Tooth Development in Edentulous Areas of the Jaw

In addition to dental epithelium invagination, early apoptosis can be also involved in the prevention of tooth development in specific regions of the jaw. A primary example of such an area is the diastema in rodents, which is an edentulous section of the jaw located between the incisors and the first molars. In the mouse, both maxillary as well as mandibular diastema reveal several rudimentary tooth germs mesial to the first molar, thought to be remnants of ancestral premolars (Peterková et al., 1996, 2002; Turecková et al., 1996; Viriot et al., 2000). However, they stop development at the epithelial thickening or bud stages and are eliminated through apoptosis (Peterková et al., 1998, 2000; Yamamoto et al., 2005). A similar mechanism of early stage tooth germ elimination was observed in the diastema of the vole (Setkova et al., 2006). These tooth germs were proposed to be remnants of ancestral premolars, which are absent in mouse. Moreover, this rudimentary tooth germ seems to be involved in the initiation of the sequential development in mouse molars, and therefore plays a key role in tooth patterning (Prochazka et al., 2010). Interestingly, the development of rudimentary tooth germs in the mandibular diastema can be rescued by the alteration of FGF signaling (Peterková et al., 2009; Li et al., 2011). Exogenous FGF8 ligand applied to the mouse embryonic diastema using protein-soaked beads rescued vestigial tooth development (Li et al., 2011). Downregulation of FGF antagonists, using transgenic animals, exhibited a similar effect on diastemal tooth initiation and their growth progression. In *Spry2^–/–^* embryos, supernumerary teeth formed in the diastemal region because of decreased apoptosis in the vestigial primordium, in association with increased proliferation (Klein et al., 2006; Peterková et al., 2009). Supernumerary tooth development was also initiated in *Spry4*^–/–^ embryos. However, a large number of supernumerary tooth germs underwent degeneration during development, resulting in a smaller number of adult animals with supernumerary teeth (Lagronova-Churava et al., 2013).

### Contribution of Apoptosis to the Decision of Final Tooth Generation

Apoptosis can contribute to the reduction of early dental germs as well as the decrease of the final number of tooth generations in some mammals. This condition was found in the Asian house shrew (*Suncus murinus*), which is a monophyodont species where the primary tooth generation is initiated up to the early bell stage but degenerates prematurely, with functional teeth developing from the second generation (Shigehara, 1980; Yamanaka et al., 2010). Apoptosis is the likely mode of breakdown since TUNEL-positive cells are found in the primary tooth germ, located on the buccal side of secondary teeth (Sasaki et al., 2001). The first generation of teeth is also aborted in the common shrew (*Sorex araneus*) through enhanced apoptosis. In this species, however, it is proposed that the replacement tooth initiates suppression of the first generation (Järvinen et al., 2008), with unknown molecular mechanisms contributing to the slowdown of the first-generation’s growth. The induction of increased apoptosis is, however, observed during growth progression in the second tooth generation and tooth germ enlargement. It is interesting that a similar induction of apoptosis is not initiated in the mouse, where the opposite condition occurs as the first generation progresses in development while the second generation remain undeveloped, but without significant apoptosis in rudimentary tooth anlage (Dosedelova et al., 2015; Popa et al., 2019).

In reptiles, it is unclear if apoptosis contributes to the removal of non-functional, early tooth generations. In the ocelot gecko, bearded dragon and the American alligator, a null generation of non-functional tooth germs is initiated (Westergaard and Ferguson, 1986; Handrigan and Richman, 2010; Zahradnicek et al., 2012). These tooth germs are located superficially and either protrude from the oral epithelium or develop deeper in the mesenchyme. The bearded dragon is the only species where apoptosis was tested and detected in the vestigial tooth generation (Handrigan and Richman, 2010). In the gecko, apoptosis was not tested in “null generation” teeth, however, their position and developmental stage ultimately affects their fate where some teeth are expelled from the oral cavity, others are incorporated into the functional teeth, while some are absorbed into surrounding tissue (Zahradnicek et al., 2012). Apoptosis can be involved in all of these processes, which will necessitate further evaluation. In the alligator, the authors inferred apoptosis as the underlying cause of their disappearance, but also did not test any aspect of apoptosis specifically (Westergaard and Ferguson, 1986).

### Regulation of Final Tooth Size Through Apoptosis

The size of individual teeth must be precisely controlled to limit or prevent its expansion into the area of neighboring enamel organs. If overlap occurs, teeth may fuse together, inducing malfunction of teeth and/or disrupting the eruption process. Apoptosis in the outer enamel epithelium and dental lamina are thought to prevent mesial and vertical overgrowth of the tooth germ; thereby representing a key cellular process regulating the final tooth size by the limitation of size expansion after certain size of tooth germ was reached. Indeed, a large number of apoptotic cells can be observed in the outer enamel epithelium of large tooth germs in pig embryos (Buchtová et al., 2012), while few apoptotic cells are observed in the relatively narrow tooth germs of polyphyodont groups such as snakes or geckos, where distances between individual teeth are extensive compared to tooth size (Edmund, 1960; DeMar, 1972).

On the other hand, it is necessary to mention that there are species such as the monophyodont veiled chameleon where the fusion of enamel organs between adjacent teeth is part of the normal developmental process (Buchtová et al., 2013). In this group, there are almost no apoptotic cells located in the outer enamel epithelium, even in very late stages when the enamel organ is large and stellate reticulum is expanded (Landova Sulcova et al., 2020), similar to pig embryos. Nevertheless, the size of the tooth germ needs to be regulated in this case as well and proper fusion of individual layers initiated. What prevents tooth germ overgrowth and controls tooth germ size in chameleon is still unknown and will be interesting to follow up in the future.

### Silencing of the Enamel Knot Signaling Center Through Apoptosis

The enamel knot is a transient, non-proliferating signaling center essential for cusp patterning during tooth development (Jernvall et al., 1994), likely involved in the evolution of various tooth morphologies in different vertebrate species (Vaahtokari et al., 1996). More than 50 genes, including some common developmental genes such as *Shh*, *Bmp-2*, *-4*, *-7*, and *Fgf-4*, have been identified as actively transcribed in the enamel knots (Vaahtokari et al., 1996; Jernvall et al., 2000). In the mouse model, the single-cusped incisors form a single enamel knot generation (Kieffer et al., 1999), while the molars, which are multi-cusped, produce multiple generations of enamel knots (Kettunen and Thesleff, 1998). During the bud to cap stage transition, PEKs develop in molars (Jernvall et al., 1998; Cho et al., 2007), while SEKs develop at the bell stage and are thought to determine the cusp position, their final number, and promote their growth by creating folds in the dental epithelium (Jernvall and Thesleff, 2000). Tertiary enamel knots (TEKs) appear next to the enamel free areas at the cusp tips and are thought to play a role in controlling the process of enamel deposition (Luukko et al., 2003).

After fulfilling their signaling roles, enamel knots are eliminated. In the incisors, enamel knots disappear through histological reorganization (Kieffer et al., 1999; Lesot et al., 2002), with only few apoptotic cells found in the knots themselves. Apoptosis does appear at the tip of the forming incisor, but prior to the histological arrangement of the enamel knot (Matalova et al., 2004). In developing molars, on the other hand, apoptosis mediates the disappearance of the PEKs at the cap stage and SEKs at the bell stage (Vaahtokari et al., 1996; Lesot et al., 2002). In studies of murine odontogenesis, induction of the apoptotic pathway in the enamel knots involves epithelial expression of *Bmp4*, *Bmpr1a*, and *Bmpr2* (Vaahtokari et al., 1996; Jernvall et al., 1998; Shigemura et al., 2001; Nadiri et al., 2004, 2006; Svandova et al., 2018), with *Bmp4* expression being dependent on *Msx2* (Bei et al., 2004). Interestingly, despite the increase in apoptotic cell numbers as the enamel knot is eliminated, the region of the tooth does not exhibit reduction in cell mass, presumably due to rapid replacement by highly proliferating cells that surround the enamel knot (Matalova et al., 2004). Furthermore, studies have suggested that the PEK may have cellular continuity with the SEK (Coin et al., 1999), which would necessitate that some cells of the PEK escape apoptosis.

### The Effect of Apoptosis on the Tooth Crown Shaping

The folding of the inner enamel epithelium contributes to enamel cusp/ridge formation in mammals. The enamel knot itself is proposed to drive epithelial bending (Jernvall et al., 1994; Vaahtokari et al., 1996). As was mentioned above, SEKs appear quite late in development, during tooth germ transition from late cap to early bell stages, when future cusps distribution is set up (Jernvall et al., 1994; Thesleff et al., 2001). The suspected role of SEKs in tooth cusp formation was confirmed in the Tabby mutant mouse, where SEKs appear to fuse together in the molar, leading to a fewer number of tooth cusps in comparison to wild-type animals (Pispa et al., 1999).

Species-specific cusp positions are determined by signaling from the enamel knots as well (Jernvall et al., 2000), with differences in the apoptotic cell distribution observed in teeth with dissimilar morphologies. In mice, there are a large number of apoptotic cells located in the inner enamel epithelium of the PEK, with only a few situated above this area, in the stratum intermedium (Vaahtokari et al., 1996; Li et al., 2016). In gerbils, which possess lophodont molars characterized by long ridges running between the buccal-lingual cusps, most of the apoptotic cells were found in deeper enamel organ area including the stratum intermedium, while almost no apoptotic cells were located in the inner enamel epithelium (Li et al., 2016). However, it is important to mention that the aforementioned study only analyzed early developmental stages and therefore SEKs were not fully formed yet, which should be more important for tooth morphogenesis (Li et al., 2016).

Odontogenesis was also analyzed in voles, which exhibit long enamel ridges and diagonal cusp pattern similarly to gerbils. This is in contrast to mice, where crests were lost during evolution (Jernvall et al., 2000). Apoptotic cells in voles also display different distribution pattern in comparison to mice, with the increased presence of apoptotic cells in the stellate reticulum, especially above the enamel knots (Setkova et al., 2006). However, again no later developmental stages with SEKs have been analyzed yet, and therefore their involvement in specific cusp patterning cannot be confirmed or ruled out.

A specific distribution of apoptotic cells was also found during the folding of the inner enamel epithelium in reptiles, where distinct structures such as enamel ridges and enamel grooves arise. In veiled chameleon and ocelot gecko (Figure 2), apoptotic cells are located in the stellate reticulum cells individually or in small clusters immediately above the enamel ridge area (Landova Sulcova et al., 2020). In the distal teeth of chameleons, two enamel ridges are formed with two distinct clusters of TUNEL-positive cells found above each enamel ridge and central groove area between them devoid of apoptotic cells. Non-apoptotic cells adjacent to those undergoing apoptosis demonstrate altered morphology with their long axes pointing in the opposite direction (Landova Sulcova et al., 2020). Similar folding and shape alterations have been observed in cells located near apoptotic cells during epithelial morphogenesis in *Drosophila*, where surface bending was induced by localized deformation of the epithelium (Monier et al., 2015), as described in section “Apoptosis in Epithelial Invagination During Early Odontogenesis.” In the chameleon, similar apico-basal forces associated with the rearrangement of cytoskeleton were proposed to be the driving mechanism contributing to the final tooth crown shape (Landova Sulcova et al., 2020). Moreover, intercellular spaces were found to widen around apoptotic cells, especially in the folding areas (Landova Sulcova et al., 2020), which indicates possible disruptions in cell adhesion molecules and loosening of their connections with neighboring cells. Alteration of protein expression in components of adherens junction molecules such as *E*-cadherin, α-catenin, and β-catenin are associated with dying cells and to contribute to the surface deformations of epithelial cells in *Drosophila* (Monier et al., 2015). In chameleons, a similar downregulation was observed in case of Na^+^/K^+^-ATPase, acting as a signal transducer (Garcia et al., 2018), during enamel ridge formation (Landova Sulcova et al., 2020), indicating the involvement of apoptotic cells in the modifications of morphogenesis through disruption of cell–cell interactions.

### Apoptosis in Hertwig’s Epithelial Root Sheath and Epithelial Rests of Malassez

Root formation is another key developmental step necessary for proper tooth attachment to underlying bone. In mammals and crocodilians, the tooth root is tightly connected to the alveolar bone by periodontal ligaments, which ensure its stable and flexible anchorage to the jaw, called gomphosis (McIntosh et al., 2002). Tooth root development is characterized by the appearance of a structure called Hertwig’s epithelial root sheath (HERS), along which the tooth root will form (Luan et al., 2006). During root formation, the outer and inner enamel epithelium first proliferate and fuse to form HERS at the cervical loop of the developing tooth (Kumakami-Sakano et al., 2014). Interestingly, this two layer-thick protrusion of the inner and outer enamel epithelium was firstly described in amphibians. Nevertheless, in mammals and crocodilians, unlike in other vertebrate species, HERS begins disintegrating from the very beginning of root elongation (Luan et al., 2006).

The HERS is thought to play an inductive role in the formation of root dentin (Bosshardt and Selvig, 1997). After dentinogenesis, ectomesenchyme cells from the dental follicle migrate through the HERS as the sheath structure is disrupted (Cho and Garant, 1988). There is still some debate about exactly how HERS disruption occurs, whether ectomesenchymal cells play a role in this process or, HERS cells disintegrate themselves and then dental follicle cells migrate through (Yamamoto et al., 2014). Regardless of the initiating mechanism, a proportion of HERS cells are thought to undergo apoptosis (Kaneko et al., 1999), while others transdifferentiate into cementoblasts (Sonoyama et al., 2007), and others still, emigrate into the periodontal ligament and form epithelial rests of Malassez (ERM) (Hamamoto et al., 1989; Kaneko et al., 1999). The process of HERS cell disintegration is accompanied with a number of cellular processes including apoptosis (Gonçalves et al., 2008), however, the fragmentation of the sheath is not caused by apoptosis directly (Suzuki et al., 2002). Programmed cell death rather helps to clear out the rest of the HERS cells which didn’t migrate to adjacent periodontal ligament or differentiate into the cementoblasts (Kaneko et al., 1999). Even though HERS is present throughout the basal vertebrates and reptiles (Luan et al., 2006; LeBlanc et al., 2021) no sign of its disintegration has been described (with the exception of crocodilians); therefore the presence of apoptotic cells is not expected.

Epithelial rests of Malassez are a cluster of cells, found predominantly in the cervical and furcation part of the tooth root, and which undergo apoptosis in order to slowly deplenish themselves (Cerri et al., 2000). The exact function of ERMs remains unknown, and theories range from a role in cementum repair (Hasegawa et al., 2003), to prevention of ankylosis of the tooth to the adjacent bone (Lindskog et al., 1988; Cerri and Katchburian, 2005). Irrespective of their specific role, there appears to be a degree of ERM cell turnover with both proliferative and apoptotic signals having been detected in these cells (Cerri and Katchburian, 2005; Lee et al., 2012). Ultimately, the number of Malassez’s rests decreases with age in both rodents (Wesselink and Beertsen, 1993) and humans (Simpson, 1965).

### The Association of Apoptosis With Tissue Differentiation

Apoptotic pathways have been shown to regulate not only cell death but also cell differentiation, based on Caspase targeting and activation of substrates or cofactors (Fernando and Megeney, 2007). In most vertebrates, teeth are capped with enamel; a unique substance that is secreted by ameloblast cells as an organic matrix and then matures into an inorganic, mineralized tissue (Lacruz et al., 2017). The development of ameloblasts includes several stages: a proliferation phase where ameloblasts differentiate into presecretory ameloblasts and begin to synthesize the enamel matrix (Karcher-Djuricic et al., 1985; Ruch, 1985), a secretory phase where enamel matrix is actively secreted (Woltgens et al., 1987; Josephsen et al., 1990), and finally, a maturation phase when cells participate in the maturation and mineralization of the enamel matrix (Kondo et al., 2001).

Between the end of the secretory phase and the beginning of enamel matrix maturation, ameloblasts enter a transition stage (Warshawsky et al., 1981; Smith and Nanci, 1995; Robinson, 2014). Apoptosis is associated with this stage of development, when ameloblast height is decreased and the disappearance of the stratum intermedium is accompanied with hypertrophy of the papillary layer (Reith, 1970; Moe and Jessen, 1972; Kallenbach, 1974; Smith and Warshawsky, 1977; Nishikawa and Sasaki, 1995; Bronckers et al., 1996; Liu et al., 2015). About 25% of the ameloblasts undergo apoptosis at the transition stage and another 25% later, during the early maturation stage (Smith and Warshawsky, 1977), when water and protein are removed from the mineralizing matrix (Nanci, 2008). Macrophages and adjacent surviving ameloblasts remove the cell debris of dying cells (Nishikawa and Sasaki, 1995). The pattern of apoptotic cell distribution in murine ameloblasts is generally similar in incisors and in the first molar tooth germs during this period. However, apoptotic cells are also located near the enamel-free area in the mouse molar cusps (Bronckers et al., 1996). In general, apoptosis is proposed to eliminate unneeded epithelial cells and regulates the number of cells entering the differentiation during amelogenesis. Moreover, it contributes to the removal of shortened and inactive ameloblasts at the end of their existence as enamel matures and becomes essentially inorganic.

In the surrounding mesenchyme, apoptosis was also identified in odontoblasts, sub-odontoblastic regions, central pulp fibroblasts, and perivascular endothelial cells (Bronckers et al., 1996; Vermelin et al., 1996; Franquin et al., 1998). Odontoblasts are primarily tasked with the formation of dentin in the vertebrate tooth (Kawashima and Okiji, 2016). A surprisingly small number of odontoblasts are thought to undergo apoptosis. A possible scenario to explain this phenomenon is that apoptosis does take place, however, rapid phagocytosis by neighboring cells contributes to the observation of the low numbers of apoptotic cells and therefore underestimation of their actual role in dentinogenesis (Franquin et al., 1998). On the other hand, apoptosis-related molecules such as *Bcl2* can affect the differentiation of odontoblasts (Zhang et al., 2010). The proposed role of apoptosis in odontoblasts is based on the necessity to retain a certain level of odontoblast turnover, which removes aged or damaged cells and possibly stimulates progenitor differentiation into mature odontoblasts in order to maintain a pool of fully functional cells (Zhang et al., 2010).

### Role of Apoptosis in Tooth Eruption

Tooth eruption is a coordinated complex of cellular and molecular process that leads to tooth relocation through its eruptive path. Here, cell death contributes to tissue remodeling and eliminates supernumerary cell populations (Kondo et al., 2001; Moriguchi et al., 2010). Five different phases can be recognized during tooth eruption: pre-eruptive movement, intra-osseous eruption, mucosal penetration, pre-occlusal eruption, and post-occlusal eruption (Marks and Schroeder, 1996). During the mucosal penetration stage, in order to establish an eruptive pathway, the connective tissue underlying the gingiva undergoes structural changes that are dependent on apoptosis and alteration of the vasculature (Marks and Schroeder, 1996; de Pizzol Júnior et al., 2015). The aforementioned ameloblast apoptosis occurs slightly later, during the eruption stage (Shibata et al., 1995; Kaneko et al., 1997).

At the initiation of tooth eruption, the epithelium of the enamel organ fuses with the oral epithelium. In the mouse embryo, the eruption of incisors takes place around postnatal day (P)10 (Greenham and Greenham, 1977), while molars erupt around P16 (Lungová et al., 2011; Dosedelova et al., 2015). During the postnatal stages of odontogenesis in mice, the superficial region of the dental lamina undergoes degeneration, not only involving apoptosis but also fenestrations of connective tissue (Dosedelova et al., 2015). However, apoptotic cells are already dispersed through the superficial lamina at P0, before the first signs of degradation are visible (Lungová et al., 2011). Since apoptosis is known to contribute to the alteration of cell adhesions through caspases activation (Kwon et al., 2015), it is likely that these early apoptotic cells initiate the disruption of epithelial integrity in the disappearing superficial dental lamina.

In developing mouse molars, apoptotic cells were also observed in more superficial areas of the dental lamina in close proximity to the oral epithelium, where the lamina merges with the dental gingiva (Dosedelova et al., 2015). However, this population of apoptotic cells was not equally distributed through the tooth and most of them were observed in the lingual area, while tissue above the erupting tooth was shed into the oral cavity.

### Involvement of Apoptosis in the Disruption and Removal of the Dental Lamina

The dental lamina develops as an epithelial protrusion growing from the oral epithelium into the mesenchyme as a continuous structure along the jaw, including interdental sections. Deep outgrowth is especially visible in diphyodont and polyphyodont species since teeth are initiated from this structure. In polyphyodont species with lifelong tooth replacement (most reptiles, amphibians, and fishes), the dental lamina connects the tooth to the oral epithelium and is retained throughout life (Buchtová et al., 2008; Zahradnicek et al., 2012), and therefore exhibits only a few apoptotic cells located in its superficial areas (Buchtová et al., 2007). An exception to this pattern of dental lamina lies in crocodilians, which are also polyphyodont. In the American alligator, juvenile and adult dental laminae do not have a connection to the oral epithelium, while they do show continuity across tooth families despite the families themselves being separated by dentary bone (Wu et al., 2013). Conversely, a study of the Nile crocodile (*Crocodylus niloticus*), which are considered “pseudoheterodont” and have different tooth types in the jaw, revealed thinning and even physical breaks in the dental lamina between tooth types along the jaw (Kieser et al., 1993). As to whether any of the abovementioned crocodilian lamina patterns are due to apoptosis remains to be seen.

On the other hand, in monophyodont or diphyodont species (most mammals and some reptiles), the dental lamina produces either one or two generations of teeth, respectively, and subsequently degenerates (Vaahtokari et al., 1996; Buchtová et al., 2012; Dosedelova et al., 2015). In monophyodont species, apoptotic cells in the dental rudiment were few and sporadic both in mice and rats (Khaejornbut et al., 1991; Dosedelova et al., 2015), as well as in veiled chameleon and bearded dragon (Buchtová et al., 2013; Salomies et al., 2019). While previously published studies proposed that apoptosis directly contributes to the regression of the successional dental lamina or the degradation of the superficial dental lamina connected to the oral epithelium (Vaahtokari et al., 1996), only a few apoptotic cells are observed in the successional dental lamina throughout its development in monophyodont species, suggesting that this may not be the entire story. Instead, the few apoptotic cells that are found in this area may contribute to lamina degradation through the induction of senescence, reduction of proliferation and loss of progenitor cells, which can lead to decelerating of lamina growth and its aging, or to the alteration of cellular adhesions of dying cells to neighboring cells, leading to lamina disintegration in diphyodont species. This hypothesis will require further examination in species with different successional dental lamina morphologies.

In the pig, which is diphyodont and has similar dental lamina morphology to humans, the breakdown of the dental lamina occurs in the middle of the prenatal period. The traditional view has attributed this fragmentation event to apoptosis (Turecková et al., 1996; Vaahtokari et al., 1996; Shigemura et al., 1999; Shigemura et al., 2001; Setkova et al., 2007). However, the observation of a generally small number of TUNEL positive cells across species, ranging from human to squamate reptiles, puts into question the actual role that apoptosis plays in the breakdown of the dental lamina (Turecková et al., 1996; Richman and Handrigan, 2011; Buchtová et al., 2012, 2013; Dosedelova et al., 2015). This uncertainty is bolstered by sometimes perplexing previously published results (Hatakeyama et al., 2000) where the authors did not observe TUNEL-positive signal in the cells of the dental lamina in 11-week human fetus, but did observe significant immunoreactivity of several active, proapoptotic proteins. In another study, the breakdown of the dental lamina was found to be largely due to epithelial-mesenchymal transformation where the dental lamina cells are transforming into mesenchymal cells, and only the cells that do not transform undergo apoptosis, which serves as a “clearing” mechanism (Buchtová et al., 2012). Furthermore, the apoptotic cells in the disintegrating lamina were mostly found in the remnants of the lamina connected with the outer epithelium, rather than in the main body of the lamina itself. This finding may explain the rarity of apoptotic cells in the degenerating dental lamina and suggests that it may only be a contributing factor to dental lamina breakdown rather than being its main mechanism (Buchtová et al., 2012).

### Apoptosis in the Mammalian Tooth Root

The tooth root is a functionally important part of mammalian dentition. A functional root anchors the tooth within the jawbone, and facilitates blood supply and innervation of the tooth (Li et al., 2017). Roots can take many shapes ranging from a simple, single root as in the human incisors, to multifurcated roots in larger teeth such as molars (Li et al., 2017). Yet, even with the morphological complexity that roots exhibit, there is little apoptosis utilized during the shaping of these intricate structures during development (Hosoya et al., 2008; Guo et al., 2015; Chen et al., 2018; He et al., 2021), with the only apoptotic cells associated with the breakdown of the HERS layer, as previously discussed.

However, there is extensive root apoptosis associated with the transition between primary and secondary tooth generations. In diphyodont mammals, the deciduous tooth generation needs to be exfoliated in order to make room for the permanent tooth. The tooth root, which consists of mineralized tissue surrounding an organic pulp, need to be broken down before the release of the primary tooth from the jaw (Figure 3). Disruption in both of these tissues involves apoptosis, with the pulp undergoing apoptosis directly (Rodrigues et al., 2009, 2012; Qian et al., 2018), while the mineralized tissue involving recruitment of special odontoclasts cells that eventually undergo apoptosis.

Odontoclasts resorb the predentine, dentine, and cementum that comprise the structure of the root, similar to the breakdown of bone by osteoclasts, in order facilitate primary tooth exfoliation and eventual replacement by the permanent generation (Sahara et al., 1996, 1998). While apoptosis of murine osteoclasts on the bone surfaces around the tooth germ has previously been document (Vaahtokari et al., 1996), there is little information on apoptosis in odontoclasts. Apoptosis in odontoclasts was analyzed by Domon et al. (2008), where they identified apoptosis in odontoclasts through TUNEL and transmission electron microscopy (TEM). Curiously, not all of the odontoclast nuclei were found to undergo apoptosis (which meant cells themselves remained alive), with intact nuclei, degenerated nuclei, and nuclear fragments found within one multinuclear cell (Domon et al., 2008). The authors explain this phenomenon by the fact that odontoclasts likely increase their number of nuclei through cell fusion, resulting in a single cell with a mixture of nuclei from older and younger cells. However, while this explains a possible reason for the pattern of apoptosis observed, the authors do not propose a reason for apoptosis in the odontoclast cells.

We theorize that odontoclasts apoptosis is either due to normal cellular turnover, or else due to the eventual elimination of odontoclasts after they have completed their function. The latter idea is supported by the fact that apoptotic signal was only identified in odontoclasts with only three or fewer nuclei (Domon et al., 2008), while the majority of odontoclasts have ten or fewer and more than half had five or fewer (Domon et al., 1997), suggesting a progressive reduction in nucleus number and presumably their function.

## Apoptotic Molecular Signaling During Odontogenesis

**FIGURE 4:**
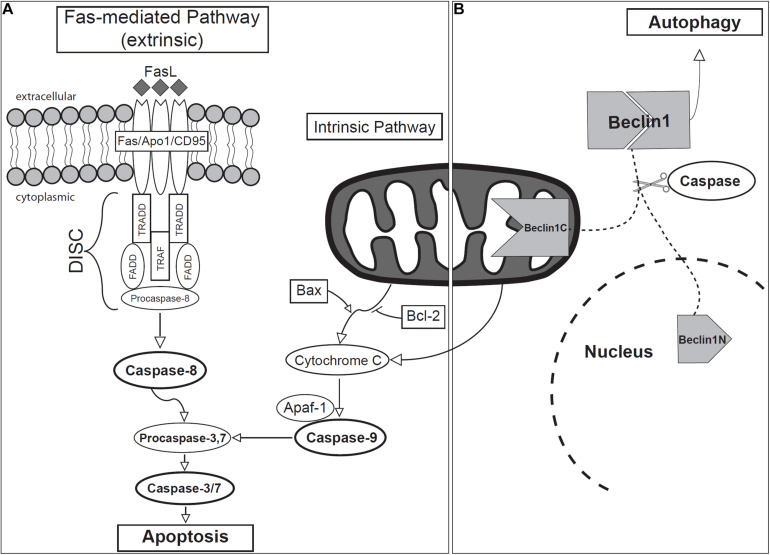
Schematic of **(A)** apoptotic and **(B)** autophagic signaling during odontogenesis.

Apoptosis may be initiated through two main molecular signaling pathways: intrinsic and extrinsic, both ultimately leading to the activation of caspases and eventual cell death (Figure 4). The molecular mechanisms involved in these pathways involve members of the following families: caspases, adaptor proteins that control the activation of caspases (e.g., TADD, FADD), members of the Bcl2 family of proteins, and members of the tumor necrosis factor (TNF) receptor (TNF-R) superfamily (Strasser et al., 2000).

### Intrinsic Apoptotic Pathway During Odontogenesis

The intrinsic pathway is activated as a response to cellular stress and involves the permeabilization of mitochondria and the release of Cytochrome *c* (Moffitt et al., 2010; Nair, 2010; Bali et al., 2013; Ghose and Shaham, 2020). Members of the Bcl2 gene family regulate the intrinsic pathway of apoptosis, acting through several proteins, both anti- (Bcl2, BclXL, Bclw, Mcl1, and A1) and pro- (Bax, Bak, Bok, Bad, Bid, Bik, Blk, Hrk, BNIP3, and BimL) apoptotic proteins, forming heterodimers to modulate each other’s function (Strasser et al., 2000; Zimmermann et al., 2001; Fan et al., 2005; Ghose and Shaham, 2020). Bax permeabilizes the outer mitochondrial membrane and facilitates the release of Cytochrome *c*, along with fragmentation of the mitochondrion itself (Youle and Karbowski, 2005). Bcl2, on the other hand, inhibits apoptosis by blocking the release of Cytochrome *c* from mitochondria (Kitamura et al., 2001; Youle and Strasser, 2008; Ghose and Shaham, 2020). After release, Cytochrome *c* subsequently forms a multi-protein complex known as the apoptosome with Apoptotic protease activating factor 1 (APAF1). The apoptosome will then activate Caspase-9 (Hakem et al., 1998), which in turn initiates a caspase cascade that leads to the activation of Caspases-3/7, and finally, apoptosis (Brentnall et al., 2013).

Bcl2 was associated with several developmental processes during odontogenesis. In human, early stage of odontogenesis revealed strong Bcl2 expression in the enamel reticulum and weaker expression in the inner and outer enamel epithelium (Kaliboviæ Govorko et al., 2010). On the other hand, Bax expression was distinct in the outer enamel epithelium (Kaliboviæ Govorko et al., 2010). Moreover, the dental lamina connecting the enamel organ with the oral epithelium was Bcl2-positive (Slootweg and De Weger, 1994). Bcl2 was proposed to maintain the viability of the enamel organ and the preserve stem cells pool in the dental lamina by preventing differentiation into squamous epithelium.

In mice, the expression of Bcl2, BclX, Bax, and Bak was found in the tooth germ at embryonic stages. Bcl2 was present in the inner dental epithelium and outer enamel epithelium. BclX, Bax, and Bak were mostly present in the enamel knot area. At the bell stage, their signal in the enamel knot was downregulated, Bcl2 was expressed in the mesenchyme in close proximity to the tip of cervical loop, while strong expression of Bak was detected in odontoblasts and stratum intermedium. All analyzed Bcl2 family members (Bcl-2, Bcl-x, Bax, and Bak) eventually exhibited downregulation at postnatal stages (Krajewski et al., 1998).

Their role in amelogenesis was described in the rat, where pre-ameloblasts displayed strong Bcl2 expression and only weak Bax-positivity (Kondo et al., 2001), suggesting that apoptosis was inhibited in these proliferating pre-ameloblasts. Co-localization of Bax and Bcl2 was revealed in the late secretory, transition, and early maturation-stage ameloblasts, however, with opposite trends in their intensity which implies an antagonistic relationship between Bcl2 and Bax during amelogenesis (Kondo et al., 2001).

Significant Fas, Fas-L, and Bax immunoreactivity was observed in the fetal dental lamina of humans, indicative of an active proapoptotic program (Hatakeyama et al., 2000). However, the expression of the pro-survival protein Bcl2 has also been identified in the dental lamina of the human tooth germ, which inhibits the apoptotic pathway (Slootweg and De Weger, 1994). Therefore, there is a question of whether the degeneration of human dental lamina is due to apoptosis or another cell death-related mechanism.

In Col2.3Bcl2 transgenic mice, in which human Bcl2 was overexpressed in odontoblasts, apoptosis rates were reduced, and differentiation was inhibited in incisors and molars compared to wild-type animals, which resulted in decreased dentin thickness and mineral density (Zhang et al., 2010). An opposite effect of Bcl2 was reported in the osteoblasts of Col2.3Bcl2 animals, where it facilitates differentiation through an up-regulation of Cbfa-1, Osterix, and Wnt/β-catenin (Pantschenko et al., 2005; Zhang et al., 2007).

### Extrinsic Apoptotic Pathway During Odontogenesis

The extrinsic pathway involves extracellular signaling and activation of transmembrane death receptors belonging to the TNF receptor superfamily [e.g., Fas (CD95/APO-1), TNFR1, DR1, DR2] (Debatin and Krammer, 2004; Lavrik et al., 2005; Nair, 2010). The Fas ligand (Fas-L) is the best-characterized molecular trigger of apoptosis (Schmitz et al., 2000). Fas itself is a cell surface glycoprotein found in most tissues and mediates apoptotic signals into the cytoplasm (Yoshioka et al., 1996; Chen et al., 1999; Nagata, 1999). Upon binding of the corresponding ligand, the Fas receptor undergoes oligomerization, forming a death-inducing signal complex (DISC), through recruitment of the cytosolic adaptor molecules such as TNFR1, Fas-associated death domain (FADD) and TNFR1-associated death domain (TRADD) (Chang and Yang, 2000; Siegel et al., 2000). FADD and TRADD then recruit and active Caspase-8, which may further activate other caspases leading to apoptosis (Susin et al., 1999; Bali et al., 2013). The N-terminal of procaspase-8 binds and activates other downstream caspases, such as Caspase-3, -4, or -7 (Muzio, 1998; Susin et al., 1999; Bali et al., 2013), inducing apoptosis.

#### Fas Receptor Function During Odontogenesis

Cells expressing Fas and FasL positive cells are primarily expressed in odontogenic epithelia (Hatakeyama et al., 2000; Kumamoto et al., 2001; Lee et al., 2012). In human patients, Fas expression was found to be more prominent in the inner and outer enamel epithelium and dental lamina than the stratum intermedium and stellate reticulum (Kumamoto et al., 2001). Similarly, Lee et al. (2012) identified expression of Fas and FasL in odontogenic epithelial cells, cementoblasts, dental follicle cells, and osteoblasts of human patients. Moreover, through co-culture experiments, they found that the Fas–FasL pathway drives apoptosis in odontogenic epithelia, ameloblasts, HERS, and ERM cells through interaction with cells of ectomesenchymal origin, dental follicle cells, and cementoblasts (Lee et al., 2012). In the human fetus of 11 weeks, Fas-positive cells were identified in the dental lamina, inner enamel epithelium, and cells of the bilateral enamel organ cusps (Hatakeyama et al., 2000). Interestingly, despite the expression of genes known to be in the apoptotic pathway, the authors describe TUNEL-positive cells as being extremely rare in the tooth germ. The high expression level of Bcl2 and weak expression of Bax led the authors to propose that Fas-mediated apoptosis was inhibited by Bcl2 in the developing human tooth germ (Hatakeyama et al., 2000).

#### Contribution of Caspases to Odontogenesis

Caspases are the key molecular mediators of apoptosis (Chang and Yang, 2000). They constitute a family of intracellular cysteine proteases that are initially produced as inactive zymogens (procaspases) and are subsequently activated through cleavage or dimerization, depending on their role (Boatright and Salvesen, 2003). As in many other gene families, the caspase family is greatly expanded in vertebrates. The human genome, for example, encodes fourteen Caspases, divided into several functional subfamilies (Pop and Salvesen, 2009). Initiator Caspases (2, 8, 9, and 10), which are involved in upstream regulatory events, have domains that bind directly to adapter molecules and are activated first. Effector or executioner Caspases (3, 6, and 7) are influenced by the inducing factors and are responsible for events directly associated with the cellular breakdown during apoptosis (Pop and Salvesen, 2009).

Caspases-8 and -9 activate effector caspase group members involved in the extrinsic and intrinsic apoptotic pathways respectively (Marzetti et al., 2012; Tummers and Green, 2017). Caspase-8 binds to the death receptor-associated FADD, forming the DISC (Fu et al., 2016; Hughes et al., 2016) and because of its binding to the complex, Caspase-8 undergoes limited proteolysis and activation. Caspase-9 is bound and activated by the apoptosome (Wu C.C. et al., 2016). Both caspases then activate downstream effector Caspases-3 and -7 (Okai et al., 2012; Wu C.C. et al., 2016; Tummers and Green, 2017), leading to cleavage of the host cell DNA, cytoskeletal scaffold protein, and the nuclear membrane (Nicholson and Thornberry, 1997; Chang and Yang, 2000).

In the murine molar, activated Caspase-3, Caspase-7, and Caspase-9 were detected in the PEK and associated with the intrinsic apoptotic pathway function (Shigemura et al., 2001; Svandova et al., 2018). Furthermore, activation was nuclear, agreeing with previous assertions that nuclear localization is associated with an apoptotic role for these enzymes (Faleiro and Lazebnik, 2000; Fischer et al., 2003; Kamada et al., 2005).

Caspase-3-positive cells in the developing tooth correspond to cells that are TUNEL-positive, indicative of apoptosis in the superficial-most layer of the dental epithelium at the initiation stage, the dental lamina throughout tooth germ development, PEK, SEK, and tips of the prospective cusps after the bell stage and into the cap stage (Shigemura et al., 2001; Nair, 2010). Casp3 knockout mice show abnormal morphology in the early bell stage tooth germ due to disruption of apoptosis (Matalova et al., 2006). This might be due to a longer persistence of the PEK cell population. Similar inhibition of apoptotic cell death in the PEK was observed in *Apaf1* and *Casp9* knockout animals, however, no observable phenotypic changes were seen in the tooth germ (Setkova et al., 2007).

Caspase-9, an initiator of the intrinsic pathway has previously been reported to be essential for apoptosis in the PEK (Setkova et al., 2007). While *Caspase-9* deficiency diminishes apoptosis (Kuida et al., 1998), the reduction does not produce an aberrant phenotype in the PEK (Setkova et al., 2007). Caspase-3 activation has also been detected in apoptotic cells of the molar PEK (Shigemura et al., 2001), however, *Casp3* deficient animals do not display significant abnormalities in early odontogenesis either (Matalova et al., 2006). Activated CASP7 was also detected in apoptotic cells of the PEK but exhibited a much wider expression pattern in comparison to caspase-3 (Svandova et al., 2018), suggestive of partially distinct roles for the two enzymes (Chandler et al., 1998; Walsh et al., 2008; Nakatsumi and Yonehara, 2010). Moreover, explant cultures of E13.5 mouse teeth with Z-VAD-fmk, a specific caspase inhibitor, revealed only little microscopic alterations or cusp pattern change, with the exception of more crowded cells in the enamel knot (Coin et al., 2000). Interestingly, expression of *Shh*, *Msx2*, *Bmp2*, and *Bmp4* (enamel knot-specific transcription factors) were downregulated in the persistent enamel knots in these experiments (Coin et al., 2000). Additionally, all three knockout animals (*Casp3*, *Apaf1*, and *Casp9*) exhibited normal final tooth shape in those that survived into adulthood (Matalova et al., 2006; Setkova et al., 2007). The effects of *Caspase-8* loss could not be studied since the *Casp8* knockout mice die before the development of the enamel knot (Varfolomeev et al., 1998).

### MicroRNAs Regulating Apoptosis During Tooth Morphogenesis

MicroRNAs (miRNAs) are naturally occurring small non-coding single stranded RNAs about 19-25 nucleotides that also regulate apoptosis during odontogenesis. Stem-loop sequence ssc-miR-133b and its target gene *Myeloid cell leukemia 1* (*Mcl-1*), a member of antiapoptotic BCL2 family protein, are key regulators of mitochondrial homeostasis, which were shown to control transitory apoptotic processes during tooth development in miniature swine (Li et al., 2018). ssc-miR-133b/Mcl-1 signaling transmitted from the mandible exosome regulate the endogenous mitochondria-linked apoptotic process during premolar development (Li et al., 2018). ssc-miR-133b is localized in both the dental epithelium and enamel knots in premolars, with stronger expression seen in the dental mesenchyme compared to the epithelium. When ssc-miR-133b was overexpressed in the premolar primary dental mesenchymal cells using lentivirus, *Mcl-1* was the only gene found to be downregulated. *Mcl-1* shares a common expression pattern with ssc-miR-133b in early tooth development and has been shown to be the downstream target of ssc-miR-133b. Over-expression of ssc-miR-133b induced the expression of the endogenous apoptotic effector *Caspase-1, 3, 7*, and *9*, whereas ectopic expression of *Mcl-1* inhibited this process, rescuing the endogenous mitochondria-related apoptotic process (Li et al., 2018).

Moreover, specific expression of miR-206-3p was observed in the dental epithelium and condensed mesenchyme of E13 mice (Neupane et al., 2020). At E14, its expression was highly restricted in the epithelium including inner enamel epithelium, outer enamel epithelium, and enamel knot. During later stage at E15, expression was localized to the inner and outer enamel epithelium. Expanded expression of miR-206-3p was also observed in the dental mesenchyme at E14 and E15 stages. The role of miR-206-3p in tooth development was tested using *in vitro* organ cultivation of embryonic tooth with an inhibitor or mimic of miR-206-3p (Neupane et al., 2020) where miR-206-3p was shown to control tooth size by regulating cell dynamics. Apoptosis was induced in both epithelium and mesenchyme following inhibition of miR-206-3p during *in vitro* organ culture. Increased apoptosis was also seen in the enamel knot suggesting its role in crown morphogenesis. miR-206-3p was also shown to regulate tooth development through the WNT pathways. The expression pattern of miR-206-3p was similar to *ß-catenin, Lef1, Pitx2*, and *Wnt3* (Sarkar and Sharpe, 1999; Kratochwil et al., 2002; Liu et al., 2008, 2013) and loss of miR-206-3p resulted in upregulation of the ß-catenin expression, whereas downregulation of *Axin2, Fzd7*, and *Wnt3* was observed in cultures using a mimic of miR-206-3p.

## Dysregulation of Apoptosis in Mouse Mutants of non-Apoptotic Genes Contributes to Disruption of Odontogenesis

The generation of genetically manipulated mouse models has become the gold standard of modern genetic research, and the field of apoptosis is no exception. However, previous research has only identified a few of the genes in the apoptotic signaling pathways, which play a key role in odontogenesis and lead to disruption of tooth formation. It is necessary to highlight that many of the genes from the apoptotic signaling cascades have not been knocked down with the aim of evaluating odontogenesis, and full understanding of their importance is still awaiting discovery. Therefore, here we also review genes that play a role in tooth morphogenesis where apoptosis contributes to the mutant phenotype. Furthermore, direct or indirect involvement of these genes in the intrinsic or extrinsic apoptotic signaling pathways still remains a mystery and will be necessary to evaluate in future.

### Mouse Models With Reduced Apoptosis

Alterations in the expression of Sprouty (Spry) genes can lead to the development of supernumerary cheek teeth anterior to the first molar by reviving the large, diastemal rudimentary tooth buds (Klein et al., 2006; Peterková et al., 2009). In wild-type mice, *Spry2* and *Spry4* are expressed in the epithelium and mesenchyme, respectively, with apoptosis repressing the formation of the rudimentary tooth buds in the posterior section of the diastema (Peterková et al., 1996; Viriot et al., 2000; Klein et al., 2006). In wild-type embryos, apoptotic cells were abundant in the anterior part of the dental epithelium and in the enamel knot at the tip of rudimentary tooth bud, whereas in *Spry2^–/–^* mutants, fewer apoptotic bodies were observed (Peterková et al., 2009). The absence of *Spry2* alters the equilibrium between growth activators and inhibitors, causing increased levels of FGF signaling leading to downregulation of apoptosis and increased proliferation in the diastemal rudimentary bud. Hyperplasia in the dental epithelium then induces the formation of supernumerary tooth primordia (Peterková et al., 2009). In *Spry2^+/–^;Spry4^+/–^* mice, the number of incisors in the upper jaw is increased due to the subdivision of the gene expression domains followed by subdivision of the single embryonic incisor anlage (Charles et al., 2011). These mice also exhibited a significantly smaller number of apoptotic elements located throughout the epithelium at E12.5 and in the central region of the incisors at E13.5, as compared to wild-type. Although the amount of cell death at E14.5 is similar in both wild-type and *Spry2^+/–^; Spry4^+/–^*, there is an alteration in the localization of the apoptotic cells, with a concentration of cells along the central axis of the mutant incisors causing the septum to separate into two dental papillae. One of possible explanation is that silencing of *Spry2* decreases phosphorylation of pro-apoptotic protein Bcl2-antagonist of cell death (BAD) similar to what was found in cell lines (Edwin and Patel, 2008), however, conformation of this mechanism *in vivo* remains to be confirmed.

Additionally, *Sostdc1*, a putative BMP antagonist (otherwise called ectodin, *Usag1*, or *Wise*), is expressed only in the dental mesenchyme and has been shown to regulate the size and placement of enamel knots and consequently to affect general morphology of mouse molars (Kassai et al., 2005). *Sostdc1* knockout mice develop ectopic teeth in the location of the vestigial premolar primordium and in the incisor region (Kassai et al., 2005; Murashima-Suginami et al., 2007). Specific lack of apoptosis was observed in the E14 *Sostdc1*-deficient incisor epithelium, within the enamel knot (Munne et al., 2009). Increased apoptosis was seen in a distinct population of cells within the dental epithelium of the wild-type incisors at the site where the extra incisors of *Sostdc1* mutants develop (Munne et al., 2009), which was comparable to other studies reported on multiple rudimentary teeth due to altered apoptosis (Turecková et al., 1996; Peterková et al., 2002).

Mice overexpressing *Ikkß* (an essential component of the Nuclear factor-kappa B (*NF-kB*) signaling) under the keratin 5 promoter developed supernumerary incisors from the embryonic epithelium. The mechanism behind the stimulation of ectopic odontogenesis was found to be reduced apoptosis and increase proliferation in the *K5-Ikkß* mice compared to wild-type (Blackburn et al., 2015). Furthermore, while *Spry2/4, Wise, and Lrp4* mutants developed ectopic incisors in both maxillae and mandibles (Ohazama et al., 2008; Munne et al., 2009; Charles et al., 2011), *K5-Ikkß* mutant mice only developed supernumerary incisors in the mandible, lingual to the endogenous incisors (Blackburn et al., 2015). Since apoptosis can be mediated by inactivation of BCL2 through IκB kinase (IKK)-dependent phosphorylation, its inhibition may also be associated with alteration of BCL2 activity in *Ikkß* mutant mice (Bodur et al., 2012). Interestingly the formation of a secondary pair of incisors lingual to the primary pair may be an ancestral trait in Glires, the mammalian clade that consists of rodents and lagomorphs (rabbits, hares, and pikas) (Asher et al., 2005). The development of two rows of maxillary teeth may still be observed in extant Lagomorphs but is thought to be inhibited through apoptosis in wild-type rodents (Asher et al., 2005; Munne et al., 2009). Therefore, it would appear that the *K5-Ikkß* mice might be recapitulating an ancestral trait.

### Mouse Models With Increased Apoptosis

The homeobox gene *Msh Homeobox 1* (*Msx1*) plays a significant role during tooth morphogenesis acting between the dental mesenchyme and epithelium. In *Msx1* null mutants, condensation of dental mesenchyme is inhibited at the bud and cap stage (Satokata and Maas, 1994), accompanied by a significant increase of apoptotic cells within the central portion of the dental epithelium (Han et al., 2003). In line with this finding, *Nestin cre;Bmpr1a* mutant mice display several tooth phenotypes including the arrest of maxillary and mandibular molars at the bud and cap stages, respectively (Andl et al., 2004; Liu et al., 2005), with maxillary incisors arrested before the bud stage, and normal incisor development in the mandible. *Nestin cre;Bmpr1a* mutant embryos also exhibited bilateral CL/P, which along with arrested tooth formation, is associated with elevated apoptosis compared to wild-type animals.

During amelogenesis and dentinogenesis, *Dentin matrix protein 1* (*Dmp1*) plays a protective role for odontoblasts and ameloblasts in the pro-apoptotic environment (Rangiani et al., 2012). *Dmp1^–/–^* mice develop hypophosphatemia, defects within dentin layer, expansion of pulp cavity and root canal (Ye et al., 2004, 2005; Feng et al., 2006). These effects can be secondarily caused by the indirect role of phosphate. To test the role of DMP1 and phosphate homeostasis during tooth development, *Dmp1*-null mice were crossed with *Klotho*-deficient mice (Rangiani et al., 2012). While *Dmp1^–/–^* mutation caused a minimal effect on enamel formation, *Dmp^–/–^/kl/kl* compound deficient mutants showed extremely severe enamel phenotype with little mineralization. This severe enamel defect in double deficient mutants is directly related to ameloblast apoptosis. Upregulation of apoptosis induced ectopic ossification, causing the formation of an ectopic mineralized matrix inside the pulp root canal proving the antiapoptotic role of DMP1 in hyperphosphatemia (Rangiani et al., 2012).

A similar protective, antiapoptotic role may also be played by Enamelin, a secreted glycoprotein that is critical for dental enamel formation. In *Enam* haploinsufficient and *Enam* null mice, extensive ameloblast apoptosis was found at all stages of enamel development (Hu et al., 2011). Staining for apoptosis in secretory-stage ameloblasts at P5 and in modulating ameloblasts at P7 and P9 in the maturation stage revealed dramatically higher cell death in the maxillary first molars of the *Enam* null mice than in heterozygous or wild-type animals (Hu et al., 2011). Additionally, the effect of Enam deficiency appears to be localized within the tooth germ as Enam null mice exhibited extensive apoptosis near the cusp tips and on the mesial cusp slopes of maxillary first molars, while ameloblasts near the cervical margin did not seem to be affected (Hu et al., 2011). Although the direct reason for the enamel pathology in *Enam* mutants is not understood, the authors propose that it may be associated with the formation of a smaller enamel layer due to *Enam* insufficiency, so that ameloblasts have a smaller surface to rest on, causing overcrowding and stress to the cells.

*Nuclear factor I-C* (*Nfic*) mutant mice develop short roots with aberrant odontoblasts during tooth development (Zur Nieden et al., 2003). *Nfic* knockout mice showed induced apoptosis in pulp cells, associated with upregulation of Caspase-8 that can cleave and activate Procaspase-3. The expression of Caspase-3 and Bcl2, which is anti-apoptotic, were also upregulated in the primary pulp cells of *Nfic* mutants compared to wild-type pulp cells (Lee et al., 2009). In addition to increased cell death in the pulp, *Nfic* mutants also exhibit preodontoblastic cells in the subodontoblastic layer, and aberrant odontoblasts trapped in abnormal dentin. Furthermore, *Tgfβ1* and *Tgfβ3* were upregulated in the *Nfic*-deficient primary pulp cells (Lee et al., 2009). Incidentally, *Tgfβ1* has also been associated with apoptosis in ameloblasts (Tsuchiya et al., 2009).

Another odontoblasts-related phenotype was found in association with a 4-bp deletion mutation in the *Distal-Less Homeobox 3* (*Dlx3*) gene, which is etiologic for most cases of Tricho-dento-osseous syndrome; a disorder characterized by abnormalities in the thickness and density of bones and teeth. When a mutant *Dlx3* copy was expressed in a transgenic mouse, the authors observed decreased dentin formation, increase in the size of the non-mineralized pulp chamber, as well as disruption of odontoblast cytology, including disrupted polarization and a reduction in overall number. Transgenic mice also exhibited curvature of the mandibular and maxillary incisors, along with a significant reduction in size. These findings led the authors to hypothesize that the mutation in *Dlx3* likely resulted in the disruption of odontoblasts cytodifferentiation, which consisted of polarization and development of the protein synthetic and secretory apparatus (e.g., the endoplasmic reticulum). Inhibited endoplasmic reticulum disrupts secretion and accumulation of dentin matrix proteins, causing stress to the odontoblasts and triggering apoptosis (Choi et al., 2010).

A different member of the homeodomain family of transcription factors, *Distal-less homeobox2* (*Dlx2*), is expressed in the cementum layer and targeted null mutation of this gene cause disruption of tooth development in mice (Thomas et al., 1997). A transgenic mouse overexpressing *Dlx2* in neural crest cells demonstrates tooth abnormalities like incisor cross-bite, shortened tooth roots, increased cementum deposition, periodontal ligament disorganization and osteoporotic alveolar bone (Dai et al., 2017). A gain of function mutation of *Dlx2* resulted in increased apoptosis in the dental germ and alveolar bone in E13.5 embryos. This is correlated with the upregulation of *Tgfßr1*, *Tgfßr2*, *Smad4*, *Msx2*, and *Sox9* expression in the dental germ. Upregulation of *Msx2* was also seen in the epithelium of the mutant with *Dlx2*-overexpression.

*Ectodysplasin A* (*Eda*) gene plays a role in ectodermal-mesenchymal interactions and mutation leads to a genetic syndrome X-linked hypohidrotic ectodermal dysplasia (XLHED) in humans. Tabby syndrome in the mouse is homologous to XLHED in humans exhibiting inborn defects in tooth number, shape, and size (Grüneberg, 1971; Blecher, 1986; Ferguson et al., 1997; Srivastava et al., 1997). In mice, *Eda* mutation suppresses *NF-kB* signaling that strongly increases apoptosis in developing ectodermal derivatives (Schmidt-Ullrich et al., 2001). Increased apoptosis is observed in mesial and distal dental epithelium in Tabby mutants compared to wild-type mice. Increased apoptosis is a secondary effect caused by defective segmentation of dental epithelium in the mice with *Eda* mutation (Boran et al., 2005).

*Vangl2*, a PCP component, is expressed in the cell membranes of inner epithelium and enamel knots of the bell stage mouse molars. Loss of VANGL2 activity causes both disruptions of cell alignment and increased apoptosis during tooth morphogenesis in mice, while *Vangl2 Lp/Lp* mutation is lethal (Montcouquiol et al., 2003). Downregulation of *Vangl2* by siRNA in *in vitro*-cultured tooth germs and their subsequent kidney transplantation revealed reduction of tooth germs growth and failed folding of inner dental epithelium (Wu Z. et al., 2016). The cellular mechanism responsible for the *siVangl2* knockdown phenotypes was identified to be a significant increase in cell death in the inner dental epithelium.

*Lymphoid enhancing factor 1* (*Lef1*) is expressed in tooth bud during mouse embryogenesis and targeted inactivation of *Lef1* induces pleiotropic phenotype impairing development of teeth. Although tooth development is initiated in these mutants, their growth is arrested at the late bud stage prior the formation of a mesenchymal dental papilla (van Genderen et al., 1994). In *Lef1* null mutants, increased apoptosis is seen within the dental epithelium, which explains the failure of the developmental transition of the tooth germ from the bud to the cap stage (Sasaki et al., 2005). The critical cell mass required to support the epithelial folding during tooth morphogenesis is affected by the significant increase in cell apoptosis with the dental epithelium.

Throughout development, ameloblasts go through several stages of development from pre-ameloblasts that are undifferentiated up to the maturation stage where mineralization of the organic matrix occurs (Bei, 2009). Throughout all these stages, ameloblasts exhibit apoptotic signals, with the highest levels detected at mature stages (Smith, 1998; Bronckers et al., 2000). Ameloblast apoptosis has been associated with the upregulation of *Transforming growth factor beta 1* (TGFβ1) in the maturation-stage enamel organ in mice (Tsuchiya et al., 2009). TGFβ1 is a growth inhibitor that has been linked to apoptosis in epithelial tissues (Foghi et al., 1998; Rosfjord and Dickson, 1999; Francis et al., 2000; Moriguchi et al., 2010). In ameloblasts, TGFβ1 induces stress-response genes which lead to a shift in the Bcl2| Bax ratio in favor of Bax, and thus induce apoptosis through the intrinsic pathway (Tsuchiya et al., 2009). Additionally, there is evidence for the function of the extrinsic pathway earlier in development, where Caspase-6 (executioner) and Caspase-8 (initiator) expression was increased in pre-ameloblasts as compared to secretory ameloblasts (Liu et al., 2015). Fas-mediated apoptosis was also identified in mature ameloblasts of rat incisors (Nishikawa, 2002). Thus, there is evidence of both intrinsic and extrinsic factors at play in the apoptosis of ameloblasts, with the extrinsic pathway functioning at earlier stages, and intrinsic pathway at later stages of maturation.

Once tooth formation is complete, apoptotic cells are also involved in the breakdown of the oral epithelium above teeth in order to facilitate eruption (Moriguchi et al., 2010; Dosedelova et al., 2015). In the investigation of P8–P15 rat pup molars during eruption, Moriguchi et al. (2010) found that apoptosis occurred at eruption sites and was suggested to be closely involved in the formation of eruption passages due to the keratinization of epithelial cells. Molecular analysis revealed that the apoptosis at eruption sites was closely associated with the Tgfβ signaling pathways. Immunohistochemical analysis of TGFβ-receptor 1, TGFβ inducible transcription factor 1 (Tieg1), NADPHoxidase 4 (Nox4), Cytochrome c, and Caspase-3 led to the following proposed model: TGFβ elevates the *Nox4* mRNA level via Smad and Tieg1, and reactive oxygen species induced by Nox4 lower the mitochondrial membrane potential, release Cytochrome c, and activate Caspase-3, which leads the cell to apoptosis.

## Autophagy During Odontogenesis

Autophagy is a lysosome-mediated cellular remodeling mechanism proposed to participate in the differentiation of odontoblasts and ameloblasts (Yang J.W. et al., 2013). Autophagy plays an important role in maintaining cellular function by removing debilitated cellular components that accumulate during cell aging (Klionsky and Emr, 2000; Vellai, 2009; Mizushima and Levine, 2010; Rubinsztein et al., 2011). In addition to its more traditionally recognized role, autophagy has also been associated with apoptosis (Wirawan et al., 2010). During odontogenesis, autophagy was confirmed through the expression of *Atg5 – Atg12*, *Microtubule-associated proteins 1A/1B light chain 3B* (LC3), as well as through transmission electron microscopy, via observation of autophagic vacuoles (Yang J.W. et al., 2013). *Atg5* and *Atg7* knockout mice do not exhibit significant abnormalities, however, deficiency in their signaling could be compensated for by different authophagy signaling or even apoptosis (Nishida et al., 2009). The key role of Atg5 was revealed by comparison of triple *Atg5/Bax/Bak* mutant mice with double *Bax/Bak* mutant animals (Arakawa et al., 2017). However, only limb and brain phenotype was analyzed and odontogenesis will need further evaluation.

LC3 is one of the commonly used markers for autophagosomal compartments in cells (Kabeya et al., 2000; Terman and Brunk, 2005; Terman et al., 2007). In developing mouse molars from E13.5 to P 15.5, LC3 expression was found in the enamel organ, dental papilla, ameloblasts, odontoblasts, dental follicle cells, HERS cells, stratum intermedium, inner and outer enamel epithelium, cervical loop, stellate reticulum, periodontal ligament, and PEK (Yang J.W. et al., 2013). In addition to localization of LC3 in odontogenic tissues, Yang et al. also found partial colocalization with TUNEL signal in the PEK area, in cells of the outer enamel epithelium, stratum intermedium cells next to the enamel knot at E16.5, the stellate reticulum, and stratum intermedium during the postnatal period (Yang J.W. et al., 2013), indicating close connection between apoptosis and autophagy during odontogenesis.

In a follow-up study from the same group, *Beclin1* (*Bcl2-interacting protein-1*) was considered as a critical player in the bridge between autophagy and apoptosis (Wirawan et al., 2010). In the developing first molar of the mouse mandible, *Beclin1* was localized to the inner and outer enamel epithelium, PEK, stellate reticulum, dental lamina, stratum intermedium, as well as differentiating ameloblasts and odontoblasts (Yang J. et al., 2013). *Beclin1* is the mammalian homolog of yeast *Atg6* gene and is involved in the autophagy activating pathway and autophagosome formation (Cecconi and Levine, 2008). The cleavage of *Beclin1* by Caspases plays a key role in the transition from autophagy to apoptosis (Wirawan et al., 2010). Caspases-3, -7, and -8 can cleave Beclin1 protein into N-terminus and C-terminus–containing fragments, which prevents the autophagic pathway. Beclin1-c then localizes to mitochondria, where it induces the release of Cytochrome *c* and initiates the intrinsic apoptotic pathway (Wirawan et al., 2010; Figure 4). Importantly, overexpression of full-length Beclin1 does not affect apoptosis (Boya and Kroemer, 2009), indicating that Caspase-mediated cleavage of Beclin1 is essential for the transition of the autophagic program toward apoptosis.

In summary, at the early embryonic stages, LC3 was detected in the dental epithelium as well as the dental papilla, while Beclin1 was mainly expressed in the dental epithelium of the developing tooth germ (Yang J. et al., 2013; Yang J.W. et al., 2013; Zhang and Chen, 2015). At the bud stage, LC3 was expressed in the epithelial bud and the mesenchyme condensed around it, while Beclin1 was mainly localized to the epithelium bud facing the oral cavity and in the tip of the tooth bud. During the cap stage, the expression pattern of Beclin1 and LC3 were similar, covering the inner and outer enamel epithelium, the cervical loop, and the PEK region facing the mesenchyme (Zhang and Chen, 2015). Overlap in the localization of Caspase 3, Beclin1 and LC3, in the PEK at the cap stage supports the notion of Beclin1 functioning as the molecular basis of crosstalk between apoptosis and autophagy during several stages of odontogenesis (Yang J. et al., 2013; Zhang and Chen, 2015).

## The Involvement of Apoptosis in Various Cellular Events During Odontogenesis – Future Directions

**FIGURE 5:**
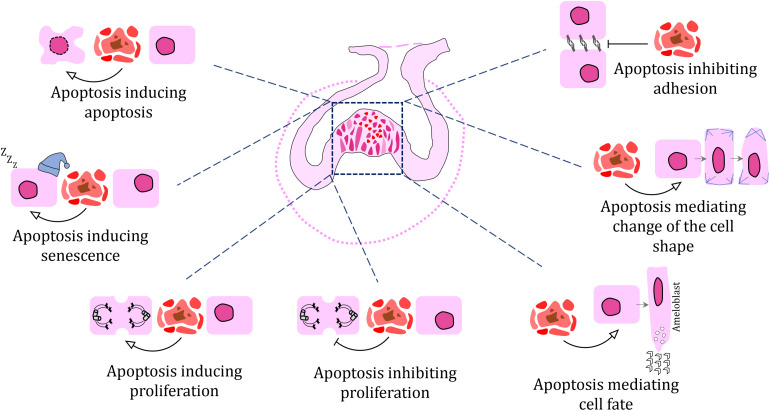
Schematic of proposed cellular processes affected by apoptosis during odontogenesis as visualized at cap stage of molar tooth germ.

To date, odontogenic research related to cell death has focused mostly on the distribution of dying cells or the expression pattern of apoptosis-related molecules. However, it seems that apoptosis can play an active role in morphogenesis as well affecting surrounding tissues by modifying numerous cellular processes (Figure 5). Not much is known about the involvement of the various non-autonomous effects of apoptosis on odontogenesis but here we would like to open this topic up for discussion and propose some possible aspects, which may be interesting to pursue in future experiments.

### Inhibition of Cell Proliferation Mediated by Apoptosis

Cell populations undergoing proliferation and apoptosis often appear as mirror images of each other within a tissue, with areas of high apoptosis usually displaying low or no proliferation (Rasch et al., 2016). There can be an effect that apoptotic cells exert on cells around them through apoptosis-associated signaling, including ligand release to their environment, that inhibit proliferation in their immediate vicinity.

### Induction of Cell Proliferation Mediated by Apoptosis

Signaling centers such as enamel knots, which exhibit high apoptotic cell numbers, are characterized by the release of morphogens (Jernvall et al., 1998), which produce a gradient in dental tissue and positively change the growth rates in surrounding structures such as the cervical loop. The questions of whether proliferation is directly induced by signals from the apoptotic cells in signaling centers, and if so, through which morphogens, remain.

### Apoptosis Mediated by Apoptosis

Apoptotic cells often aggregate in large clusters, especially in the enamel knots (Lesot et al., 1996). The breakdown of enamel knots starts with the presence of only a few apoptotic cells in the area, with later expansion into neighboring cells, which also undergo apoptosis. Is the expansion of the apoptotic cell population in the enamel knot driven and induces by the first generation of a few dying cells or by their apoptotic signaling? Or is apoptosis innately predetermined in the individual cells of the enamel knot?

### Senescence Mediated by Apoptosis

Are non-proliferating cells surrounding apoptotic cells senescent? The expression of several senescence markers such as cyclin-dependent kinase inhibitor *p21* (Keränen et al., 1998) have been observed in apoptotic areas, however, direct confirmation of their senescent status has not been provided. Moreover, in enamel ridges, cells closely associated with those undergoing apoptosis will produce lipid droplets in the direction of shared cellular membranes. The accumulation of intracellular lipids can influence the regulation of fatty acid synthesis, which in turn controls cell senescence (Flor et al., 2017). However, the relationships of these processes still need to be investigated during odontogenesis.

### Cell Adhesion Changes Mediated by Apoptosis

As cells undergo apoptosis, they become detached from neighboring cells. In fact, cell adhesion changes are associated with the induction of apoptosis, with the expression of membranous complexes becoming altered (Monier et al., 2015). As cells are released from their fixed positions, surrounding cells can actively migrate or be passively translocated to new destinations, which can ultimately affect the final shape of epithelial structures.

### Apoptosis Driving Cell Polarity Changes in Neighboring Cell Populations

In areas where apoptotic cells accumulate, cells not only lose their attachment to neighbors but experience reorientation of their axes, which is accompanied by changes in cell polarity (Warner et al., 2010). These processes accompany the morphogenesis of inner enamel epithelium and complex tooth shape formation. Nevertheless, can apoptotic cells directly induce changes in cell polarity, or is this just a secondary effect of the alteration of their connection to neighbors?

### Apoptosis Inducing Changes in Cell Shape

Apoptotic cells can induce the rearrangement of cytoskeleton, causing local deformation of the apical surface of surrounding epithelial cells (Monier et al., 2015). However, it is not known if the apico-basal pulling force, which is generated by the dying cell in the dental epithelium, can be strong enough to induce invagination or cell rearrangement. This feature will need further functional proof.

### Cell Fate Directed by Neighboring Apoptotic Cells

Apoptotic cells can also induce differentiation of surrounding cells, contributing to their developmental progression and transition to the next developmental stage (Zhang et al., 2010). How directed differentiation of only certain cells occurs in dental epithelium by apoptotic cells while not affecting all surrounding cells is still unknown and will be necessary to uncover in the future.

## Conclusion

Apoptosis plays an active role in odontogenesis and apoptotic cells dynamically influence surrounding cells to trigger tissue remodeling through the regulation of cell division, cell death, cell fate, migration, cell shape, and remodeling of nearby tissues but cellular mechanisms driving these processes are mostly unknown. Next, it will be necessary to study these less expected roles of apoptotic cells signaling not only in odontogenesis but also their contribution to the regeneration of dental tissues or induction of pathological conditions in teeth.

## Author Contributions

MB, JA, MŠ, and PG-L: original draft writing. MB, JA, MŠ, and PG-L: final manuscript writing. JA and MŠ: drawing. MŠ: immunohistochemical staining.

## Conflict of Interest

The authors declare that the research was conducted in the absence of any commercial or financial relationships that could be construed as a potential conflict of interest.
